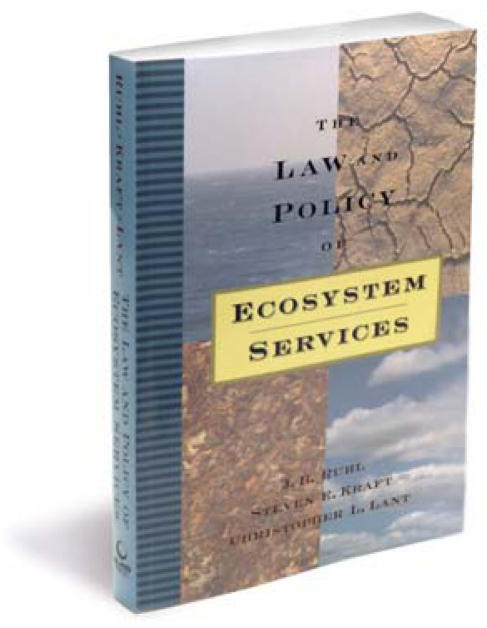# The Law and Policy of Ecosystem Services

**Published:** 2007-08

**Authors:** Robert Epting

**Affiliations:** Robert Epting is an environmental attorney with Epting & Hackney in Chapel Hill, North Carolina. He has served on the North Carolina Environmental Management Commission and has received numerous public service awards

By J.B. Ruhl, Steven E. Kraft, and Christopher L. Lant

Washington, DC:Island Press, 2007. 350 pp. ISBN: 978-1-55963-094-8, $70

Environmental law and public policy arise out of traditional conflicts between different parties interested in the use of air, light, water, and the peaceful surround for competing purposes. For example, the noxious smell of your hog waste ponds ruins the fresh air around my home. Or the shopping center you are planning to build on the banks of Community Creek will in fact cover and destroy 30 acres of wetland just upstream of our downtown.

Are the interests to be weighed in such disputes merely private, or must we also recognize—and regulate to protect—the community’s interest in the natural ecosystem services to clean air and clean water, possibly lost as a consequence?

The law of nuisance has long provided remedy for neighbors who are caused to suffer noxious effluent arising from nearby agricultural or industrial uses. And since the 1970s, federal and state clean water and clean air legislation, and indeed the entire U.S. Environmental Protection Agency apparatus whereby government permits and regulates pollution, have arisen from the idea that the economic engine of this country, running on a seemingly limitless reservoir of natural resources, must now be regulated to limit, if not prohibit, the production of goods and services by methods that cause irreversible harm to air and water resources.

The idea behind *The Law and Policy of Ecosystem Services* is that courts, legislatures, regulators, and other policy makers have traditionally been without the tools to value, or otherwise have taken too little account of the value of, ecosystem services as environmental policy is developed. And, they argue, as a result, we risk this “tragedy of ecosystem services”:

By the time scarcity alone focuses economic investment on ecosystem services, we may not have sufficient natural capital resources available in the quantities and quality demanded, and we may not be able to create enough either. The reason for the decline of ecosystem services will be a continued degradation of ecosystem functions that support natural capital resources. The degradation that ecosystem functions suffer is not likely to be fully reversible. Indeed, it is more likely that the decline of ecosystems by that point will in many cases have become irreversible.

The authors assert, therefore, that refocusing our public law and policy to value, account for, and prevent erosion and loss of vital ecosystem services, rather than weighing, minimizing, or remedying functional losses primarily to private interests, is essential if we are to recognize and protect the nature, extent, and natural importance of these resources before their loss becomes irreversible.

The authors demonstrate persuasively that natural capital and ecosystem services are not adequately recognized or valued under present law and policy, and they show how a new polity of law and regulation, exceeding that of traditional private economic interests, might be achieved.

I admit that many more expert readers will find the detailed history and analysis of the geography and economics of ecological services more familiar and useful to their understanding.

My own experience as an environmental lawyer who has traditionally represented public and private parties injured by air or water pollution, as well as public agencies with water and air pollution permits, leads me to endorse this book’s ideas and to suggest that it should be required reading for the whole range of players—from legislators and policy makers to land trust administrators and riverkeepers—whose work revolves around the natural tensions between conservation of public, natural capital and the extension of private capital. Environmental litigators also will find in this work a source of useful advantages in perspective and tactic when battling traditional opponents who argue that their noxious byproducts are merely “the smell of money,” as if natural ecosystem capital and services should always bear private capital’s avoided costs of proper waste handling.

Currently, elected officials who appoint the regulators and formulate environmental policy at the state and federal levels show little inclination to shift their attention to protect natural ecosystem capital, especially at the expense of discouraging unbridled growth, even though the work of many of their employees and subordinates suggests their ripening attention and appreciation for the importance of acknowledging, valuing, and protecting ecosystem services.

The ideas expressed in this book underscore the importance of that dutiful lot, at work to protect our natural environment, and suggest important directions and tools, useful if we are to appreciate, conserve, and avoid long-term, tragic injury to the natural capital and ecosystem services that sustain us all.

## Figures and Tables

**Figure:**